# Utility and Limitations of Using Gene Expression Data to Identify Functional Associations

**DOI:** 10.1371/journal.pcbi.1005244

**Published:** 2016-12-09

**Authors:** Sahra Uygun, Cheng Peng, Melissa D. Lehti-Shiu, Robert L. Last, Shin-Han Shiu

**Affiliations:** 1 Genetics Program, Michigan State University, East Lansing, Michigan, United States of America; 2 Department of Plant Biology, Michigan State University, East Lansing, Michigan, United States of America; 3 Department of Biochemistry and Molecular Biology, Michigan State University, East Lansing, Michigan, United States of America; University of Southern California, UNITED STATES

## Abstract

Gene co-expression has been widely used to hypothesize gene function through guilt-by association. However, it is not clear to what degree co-expression is informative, whether it can be applied to genes involved in different biological processes, and how the type of dataset impacts inferences about gene functions. Here our goal is to assess the utility and limitations of using co-expression as a criterion to recover functional associations between genes. By determining the percentage of gene pairs in a metabolic pathway with significant expression correlation, we found that many genes in the same pathway do not have similar transcript profiles and the choice of dataset, annotation quality, gene function, expression similarity measure, and clustering approach significantly impacts the ability to recover functional associations between genes using *Arabidopsis thaliana* as an example. Some datasets are more informative in capturing coordinated expression profiles and larger data sets are not always better. In addition, to recover the maximum number of known pathways and identify candidate genes with similar functions, it is important to explore rather exhaustively multiple dataset combinations, similarity measures, clustering algorithms and parameters. Finally, we validated the biological relevance of co-expression cluster memberships with an independent phenomics dataset and found that genes that consistently cluster with leucine degradation genes tend to have similar leucine levels in mutants. This study provides a framework for obtaining gene functional associations by maximizing the information that can be obtained from gene expression datasets.

## Author Summary

There remain genes with no known function even in the most well studied, model species. One common way to hypothesize gene function is based on the assumption that genes with similar expression profiles tend to have similar functions. However, using datasets and biological pathway information from the model plant *Arabidopsis thaliana* as an example, we discovered that, although genes in the same pathways are functionally related, genes in only a subset of the pathways have highly similar expression patterns. In addition, our ability to hypothesize gene functions based on expression is significantly impacted by how the dataset is processed and combined as well as the methodology used to identify genes with similar expression. Therefore, multiple datasets and methods should be tested to maximize the functional information that we can get based on similarity in gene expression.

## Introduction

With the ease of sequencing, an ever increasing number of genomes from a wide range of species are available. One major challenge is to ascribe functions to genomic features. For example, while ~70% of *Arabidopsis thaliana* genes have annotated functions [1], only ~40% of these annotations are supported by experimental evidence such as mutant phenotype or biochemical assays [2]. To increase functional information, transcriptome data have been used to develop hypotheses of gene function based on similarity of expression patterns (co-expression) with genes that have known functions [2–4]. The relationship between co-expression and functional correlation was first shown with *Saccharomyces cerevisiae* and human transcriptome data [5–8]. Subsequently, a large number of plant studies used co-expression analysis to infer gene functions [9–17]. For example, the *MYB28* and *MYB29* transcription factors are co-expressed with the glucosinolate pathway genes that they regulate [9]. Similarly, the transcription factors *CRC* and *AP1* co-express with 58 fatty acid biosynthesis genes, and *crc* and *ap1* mutants have altered fatty acid compositions [15]. More broadly, methods based on integration of multiple types of omics datasets were developed to account for different levels of regulation and to improve gene functional inferences [18–21]. In these data integration exercises, transcriptome data remain the most abundant and the most effective in capturing gene functional relationships [2,18]. Thus, analysis of gene expression results can lead to hypotheses of plant gene functions.

Despite its utility, there are known computational and biological limitations in using co-expression for gene functional inference, and these usually are not evaluated in co-expression based studies [2]. First, genes with similar expression profiles may not necessarily have related functions [22]. Second, for those genes that do have related functions, transcription patterns may not be coordinated due to post-transcriptional and other levels of regulation [23]. Third, it is also possible that they do in fact co-express, but that the co-expression criteria need to be optimized. For example, using an expression coherence (EC) measure, which is the ratio of the number of co-expressed gene pairs to the total number of gene pairs [24], only 41% of the Gene Ontology Biological Process (GO-BP) terms have higher ECs than expected by chance [25]. The 59% of pathways with low ECs may contain genes that are regulated beyond transcription. Alternatively, a more detailed exploration is required to determine how co-expression should be defined. Consistent with this, in most studies, a fixed threshold of expression similarity is used to identify pairs of co-expressed genes. Depending on the value of this threshold, the degree of co-expression might be over- or underestimated and lead to false positive or negative associations. Therefore, it is necessary to optimize the criteria used to define co-expression to increase the utility of expression data in guilt-by-association studies.

One major parameter that impacts co-expression studies is the type of dataset; it is expected that not all expression profiling experiments will be informative for revealing functional relationships between any given gene pair [26]. Most studies combine multiple datasets for gene function inference [9,25]. One advantage of this approach is the increased statistical power for establishing correlations. Small number of samples might lead to statistically unreliable connections [27]. However, the inclusion of too many samples can result in the loss of information [28], and expression datasets that are directly relevant to the underlying biological processes might be more useful in functional inference. For example, to uncover drought response pathway genes, it would be better to use a more specific, drought stress dataset instead of a collection that includes potentially uninformative experiments [13]. Other factors that impact the effectiveness of co-expression studies include the specific samples used (e.g. stress vs. developmental series), method of data transformation (e.g. fold change vs. absolute expression values), and the procedures and parameters used to define co-expression. A comprehensive study evaluating the above is needed and would be highly informative for future studies that use co-expression as a means for functional inference.

In addition to inferring functional relationships between two genes, co-expression is useful for uncovering groups of genes with related functions (referred to as clusters). Unsupervised learning methods, particularly various clustering algorithms, are among the most common approaches used to identify co-expression clusters [29]. Once the clusters are identified, functional categories such as GO can be used to evaluate what types of genes are over-represented in each cluster, and gene functions can be hypothesized based on cluster membership [30]. Although clustering and enrichment analyses are straightforward, there is no single best method [31] as there are a large number of clustering algorithms and the cluster memberships (which genes are in the same cluster) depend on many clustering variables (e.g. algorithm, distance measure and number of clusters). Because differences in parameter choice strongly influence the types of co-expression clusters obtained, it is important to perform clustering with multiple parameters rather than relying on a single method.

In this study, our goal was to maximize the information from co-expression data to improve predictions of functional associations between genes. Specifically, we asked to what extent *A*. *thaliana* genes are co-expressed in each metabolic pathway. We also explored the features of high EC pathways. Next, we evaluated the influence of dataset on EC for each metabolic pathway, the best practices in using co-expression to identify novel genes that function in a biological process, and the impact of different commonly-used clustering algorithms and parameters on the ability to identify genes that function in the same pathways. Finally, the biological relevance of cluster membership was validated using an independent phenomics dataset. Overall, we demonstrated that optimizing the use of co-expression based approaches requires consideration of the pathway of interest, expression dataset and clustering algorithm.

## Methods

### *A*. *thaliana* metabolic pathways and pathway features analyzed

*A*. *thaliana* metabolic pathways (AraCyc pathways), the genes belonging to these pathways and supporting evidence were obtained from the Plant Metabolic Network (version 8, [32]). To examine a broader set of gene function in addition to metabolism, *A*. *thaliana* Gene Ontology biological processes (GO-BPs) annotations were obtained from geneontology.org [33]. Only nuclear genes and pathways/processes with >2 genes were included in further analyses. The metabolic pathway genes were divided into two sets based on supporting evidence. The first set contained all pathway genes regardless of the types of evidence supporting the annotations (382 pathways, 5,991 genes). The second set only contained genes with experimental evidence (225 pathways, 934 genes). For the GO data, we examined 1,710 GO-BP terms covering 23,157 genes.

To determine if genes of a pathway tend to have a particular subcellular location, subcellular location information was obtained from the SUBcellular Arabidopsis consensus database (SUBAcon [34]), and a contingency table for each pathway and subcellular location was established to calculate the enrichment *p-*value (Fisher’s Exact Test). The resulting *p*-values were corrected for multiple testing [35]. To determine whether similar sets of *cis*-regulatory elements are present among genes in the same pathway, 349 position frequency matrices taken from the Cis-BP database [36] were converted to position weight matrices (PWMs) based on the *A*. *thaliana* background AT and CG frequencies (0.33 and 0.17, respectively) using the Tools for Analysis of MOtifs (TAMO) package MotifTools [37]. The PWMs were used to determine the location of motif sites in the 1kb region upstream of the transcriptional start sites of *A*. *thaliana* genes with Motility [38]. To assess the impact of post-transcriptional regulation, we used a dataset with associations between miRNAs and their target genes, downloaded from The Arabidopsis Information Resource (TAIR, [39]).

### Expression dataset and its processing

Six publicly available Affymetrix ATH1 microarray gene expression datasets used in this study include: Biotic stress: GSE5615-5616, Light: GSE5617, Abiotic stress: GSE5620-5628, Development: GSE5629-5634, Hormone: GSE39384, and Diurnal [40–43]. In addition to these, ~700 *A*. *thaliana* microarray datasets were downloaded from NASCArrays database [44]. The datasets were downloaded in either normalized form [44,45] or as unprocessed data from Gene Expression Omnibus (GEO) [46]. For the unprocessed datasets, the CEL files for the AtGenExpress data [40–42] were downloaded from TAIR [39] and quantile normalized using the Bioconductor affy package in R [47]. The Bioconductor LIMMA package [48] was used to calculate fold changes by contrasting treatment and control experiments, and the *p*–values of significant fold changes were corrected for multiple testing [35].

### Calculation of expression correlation and expression coherence

To generate the null expression correlation distribution, 500,000 gene pairs were randomly selected and their Pearson Correlation Coefficients (PCCs) were calculated using the SciPy library [49]. The 95th percentile PCC values (PCC95) in the null distributions were used as thresholds for calling the expression patterns of two genes as significantly correlated with a 5% false positive rate. Using the PCC95 values, the expression coherence (EC) score was calculated to determine the extent of co-expression among genes in a given pathway [24,25]. The EC score of a pathway is the ratio of the number of gene pairs with PCC values higher than PCC95 and the total number of gene pairs in a pathway. Thus, EC values range from 0 (no gene pair with significant expression correlation) to 1 (all gene pairs significantly co-expressed). To identify pathways with significantly higher than randomly expected ECs (high EC pathways), pathway-gene associations were randomized 100 times with the sizes of the pathways kept the same. For each dataset, a distribution of randomly expected EC values was established. For a given dataset, a pathway was defined as a high EC pathway if it had an EC score larger than the 95^th^ percentile value of the null EC distribution. The percentile of the pathway ECs in the null EC distribution was referred to as EC percentile. To assess how similar the gene expression profiles among array experiments in a dataset are, the PCC values between the experiments in a dataset were calculated and the median PCC value was used as a measure of homogeneity among the experiments within a dataset.

To evaluate the impact of similarity measures, Spearman’s rank coefficient [49], partial correlation [50] and Mutual Information (MI) [51] were used as additional similarity measures to determine pathway EC in the same way as PCC was used. Partial correlations of pathway genes were calculated with two methods: (1) a Python implementation of partialcorr function in MATLAB, which determines the correlations between residuals of linear regression, and (2) the R package corpcor that was optimized for genomic datasets [50]. MI was calculated both as normalized and adjusted with the Python scikit-learn package [51]. The adjusted MI measure accounts for impact of sample size (larger samples might lead to higher MI) and the normalized MI value was calculated by scaling MI values to between 0 and 1. Bayesian Networks (BNs) were constructed for each pathway using the bnlearn package in R [52]. Hill-climbing algorithm was used to construct BNs with options for continuous data. The transformed *p-*values (-log(*p*)) of arc strengths between nodes (genes) in BNs were used as measures of gene association strengths that are used similarly as pairwise similarity. Only the transformed *p-*values were used because they were nearly perfectly correlated with arc strengths (*r*^*2*^ = 0.9998). BNs were also constructed for randomized pathways to determine threshold *p-*values for each gene association and the thresholds were then applied to determine how many gene pairs in each pathway have above threshold arc strength *p-*values to determine pathway EC.

### Co-expression clustering

To determine the impact of the clustering algorithm on the resulting co-expressed gene clusters, we tested *k*-means [53], hierarchical clustering (hclust), *c*-means [54] and Weighted Gene Co-expression Network Analysis (WGCNA) [55] in the R environment and approximate kernel *k*-means [56] in MATLAB. Clustering parameters tested included the numbers of clusters (*k*), distance measures, and hierarchical clustering algorithms for relevant methods. Initially, we attempted to obtain the optimal *k* for clustering the stress expression dataset by obtaining the “elbow plot”. After testing 11 *k* values ranging from 5 to 2000, we realized that the selected *k* was not necessarily the best and the choice of *k* impacts clustering memberships of genes. For distance-based algorithms, three distance measures (Euclidean, radial basis function kernel and 1-PCC) were tested. For hierarchical clustering, we also explored the impact of average, complete and Ward linkage algorithms. For WGCNA, the pickSoftThreshold function was used to determine the ß values based on the scale-free topology model [55]. Commonly used clustering algorithms—such as *k*-means—are not deterministic, i.e. they may result in a local optimum solution. To evaluate whether multiple runs could result in significantly different results, we ran *k-*means, approximate kernel *k-*means and *c*-means 10 times. We refer to the similarity among 10 runs as consistency between the runs. In contrast, for hierarchical clustering and WGCNA, the co-expression cluster membership was always the same for every run.

### Assessing the overlap in pathway and cluster memberships

Fisher’s exact test was used to assess how well memberships within a cluster overlap with those in a pathway. The resulting *p*-values were corrected for multiple testing [35]. For each clustering algorithm-parameter combination, an “over-representation score” between a cluster and a pathway was defined as the -log(*q*) value where a higher score indicates a more significant degree of overlap between cluster and pathway memberships. An over-representation score ≥1.3 (*q* <0.05) was considered to be statistically significant. To account for the possibility that over-representation of some pathways is spurious we asked how often significant over-representation scores arise from randomized expression data. Specifically, the stress expression dataset was permuted to generate 15 random datasets that were used in *k*-means clustering (*k* = 5 to 2000, 10 independent runs for each *k* and each random dataset). The same approach outlined above was also used to assess how well memberships in a pathway overlap with those in a random cluster. Among 1,650 random clusters, none had a significant over-representation score with *A*. *thaliana* pathways.

To further assess if cluster membership can serve to predict pathway membership, we calculated the F measure (the harmonic mean of precision and recall) for each cluster-pathway combination. Precision is the proportion of correct predictions over total predictions; in our case it was the ratio between the number of genes in a cluster that were also found in a pathway and the total number of genes in that cluster. Recall is the proportion of correct predictions over total true positives; in our case it was the ratio between the number of genes in a cluster that were also found in a pathway and the total number of genes in that pathway. F measure was calculated for each pathway-cluster combination with an over-representation score ≥1.3.

### Using phenomics data to evaluate co-expression associations

Here we used the mutant profile data from Chloroplast 2010, a database consisting of phenotypic screening results for mutants of more than 5,000 genes [57,58] to confirm the potential functional links between genes found in the same co-expression cluster. This database includes measurements of amino acids and fatty acids as well as chloroplast morphology and photosynthetic parameters. Taking leucine degradation as an example, we expected the leucine content to be more similar between mutants of leucine degradation genes and mutants of genes found in the same co-expression cluster than to wild-type and mutants of random genes. To determine whether this was the case, we retrieved the leucine measurements (in nmol/g fresh weight) of 12 leucine annotated degradation genes, genes that were clustered with pathway genes with an over-representation score >1.3 (*q* <0.05), 1000 random genes, where homozygous T-DNA insertions were available, and 184 wild type control plants included in the Chloroplast 2010 database. Significant differences between the leucine levels of mutants and controls, which included randomly selected mutants of genes that are not in the leucine degradation pathway and wild-type plants, were identified with Mann-Whitney tests.

## Results

### The extent to which genes in pathways have correlated transcript profiles

**Fig 1:**
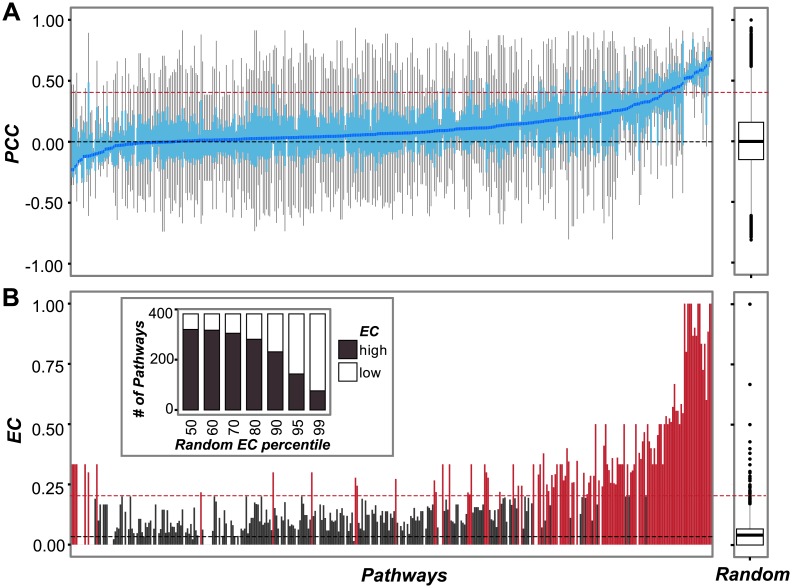
Co-expression of *A*. *thaliana* pathway genes under stress. **(A)** Boxplots of expression correlations (Pearson’s Correlation Coefficient, PCC) between pairs of genes in each *A*. *thaliana* metabolic pathway (left sub-figure) and random gene pairs (right sub-figure). The pathways are sorted based on median PCC. Light blue boxes: Interquartile range. Blue line: median PCCs. Red dashed line: the 95^th^ percentile PCC value (PCC95 = 0.41) of the random gene pair PCC distribution. Black dashed line: the median PCC of the random gene pair PCC distribution. **(B)** Bar plot indicating ECs for *A*. *thaliana* pathways (left sub-figure) and random gene pairs (right sub-figure). The pathways are in the same order as in (A). The insert graph shows the number of pathways that have significantly higher ECs than randomly expected (black) and those that are not significant (white). Different percentile thresholds on the x-axis are based on the random EC distribution (right sub-figure). The red dashed line designates the 95^th^ percentile of the random EC distribution.

To evaluate the extent to which genes with similar expression patterns have similar functions, we asked whether genes in the same *A*. *thaliana* metabolic pathway were co-expressed (see Methods). To address this question, Pearson Correlation Coefficients (PCCs) between genes in each of the 382 *A*. *thaliana* metabolic pathways in AraCyc were calculated using an expression dataset consisting of 16 different environmental conditions (referred to as the stress dataset [41]) (Fig 1A). To broadly examine groups of functionally related genes in addition to metabolic pathways, we also calculated PCCs between genes in each of the 1,710 *A*. *thaliana* Gene Ontology Biological Process (GO-BP) categories. A group of genes in an AraCyc pathway or a GO-BP is referred to as a “functional category”. The median PCC values were <0.1 for ~60% of functional categories, suggesting that many genes in the same pathway have dissimilar transcript profiles under stress conditions. To assess statistical significance and control for false positive expression correlation, the PCC values of pairs of genes in the same functional category were compared to PCC values of random gene pairs (Fig 1A). The 95^th^ percentile PCC value of random gene pairs (referred to as PCC95) was 0.41 for the stress dataset. In other words, only 5% of random gene pairs have PCC values >0.41. We used PCC95 as the threshold for calling the expression profiles of a gene pair as significantly positively correlated with a 5% false positive rate. Based on this threshold, only 19% of gene pairs within a functional category have significantly correlated expression patterns.

To determine whether some functional categories contain more members with highly correlated expression than others, we adopted the expression coherence (EC) measure, which ranges from 0 to 1 [25]. Here the "pathway EC" is defined as the proportion of pairs of genes in a pathway or GO category that have significantly correlated transcript profiles. Note that the median ECs in *A*. *thaliana* are only 0.11 for GO-BPs and 0.14 for AraCyc pathways, indicating that 50% of the functional categories have <11–14% gene pairs with significant expression correlation. Consistent with an earlier study [25], we found that genes in functional categories generally have higher ECs than groups consisting of randomly selected genes (Mann-Whitney test, *p* <2.2e-16; Fig 1B; S1A Table). In particular, 36% of the AraCyc pathways have higher EC values than the 95^th^ percentile of the random EC distribution (Fig 1B); these are defined as “high EC pathways”. Similarly, 32% of the GO-BPs have higher EC values than the 95^th^ percentile of the random EC distribution (referred to as “high EC GO-BPs”, S1A Table). One explanation for the slightly higher number of high EC pathways than that of high EC GO-BPs may be because metabolism related pathways tend to have more highly coordinated transcriptional regulation compared to other types of functional categories. Consistent with this notion, GO-BP categories related to metabolism, including metabolic pathways (GO:0008152) and its child terms, have higher median ECs (0.14 and 0.13) compared to signal transduction (GO:0007165, EC = 0.11), cell-cycle (GO:0007049, EC = 0.10) and response to stress (GO:0006950, EC = 0.08) categories (S1 Fig). Among metabolic GO-BPs, amino acid metabolism pathways (GO:0006520) have the highest median EC (0.21) among the categories we compared (S1 Fig). Overall, GO-BPs have lower ECs than AraCyc pathways (Mann-Whitney Test, *p* = 2.41e-03; S1 Fig).

The ECs for functional categories have a very wide range (Fig 1B; S1 Fig). The differences in ECs may be due to technical issues such as functional annotation quality or methodological issues such as the similarity measure used to assess co-expression. The EC differences can also be due to differences in the biological characteristics of pathways, for example, the role of the pathway, presence of common transcriptional regulatory mechanisms, and regulation at levels beyond transcription. Finally, the dataset used to calculate EC could also be a major factor. In the following sections, we assess the factors influencing ECs and identify ways to maximize ECs for functional categories. Considering false positive annotation can have a significant, negative impact in further analyses, we examined features of high EC categories and the impact of multiple factors on ECs by focusing on AraCyc metabolic pathways in the following sections.

### Influence of annotation on pathway ECs

**Fig 2:**
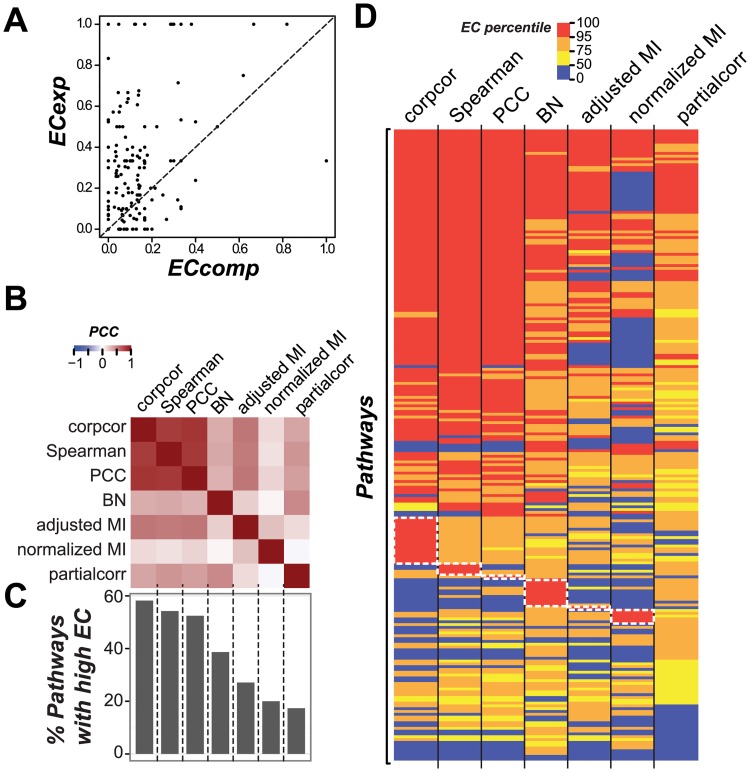
Relationship between pathway ECs, annotation quality and similarity measures. **(A)** Relationship between the EC calculated for pathway genes that are annotated based on experimental evidence (ECexp) and EC calculated for pathway genes that are annotated only based on computational evidence (ECcomp). The genes used to calculate ECexp and ECcomp do not overlap. Each dot represents one pathway. Dashed line: *y* = *x* line. **(B)** Heatmap of correlations between pathway EC percentiles calculated with: partial correlations estimated with the corpcor method, Spearman’s rank correlation coefficient (Spearman), Pearson Correlation Coefficient (PCC), adjusted and normalized Mutual Information (MI), partial correlation calculated with the partialcorr method, and transformed *p-*values of Bayesian Network (BN) (**C)** Percent pathways that have high EC using different similarity measures. **(D)** Heatmap of pathway EC percentiles calculated using different similarity measures. Color represents EC percentiles. White dotted rectangles: high EC pathways that are specific to one measure.

Computational predictions of gene function without experimental evidence can lead to false assignments to pathways, resulting in lower pathway EC values. This is particularly important because computational annotations in the Plant Metabolic Network are based on sequence similarity only [59,60]. Functional annotations made using sequence similarity based methods are estimated to have an error rate of 49% [61] and high sequence similarity does not necessarily lead to co-expression [62]. To determine whether annotation quality is a major factor influencing pathway EC, we separated pathway genes into those with and without experimental evidence. Consistent with the hypothesis that annotation quality can significantly impact pathway EC, pathways with lower ECs tended to have proportionally fewer genes with experimental evidence (PCC = 0.20, *p* = 1.53e03; S2A Fig). Pathway ECs calculated using genes with experimental evidence were substantially higher (Mann-Whitney test, *p* = 5.44e-12, median EC = 0.26) than those calculated using genes assigned to pathways solely based on computational predictions (median EC = 0.10; Fig 2A). This is consistent with the hypothesis that some annotations based solely on computational evidence are incorrect.

Although annotation quality influences pathway EC, it explains only ~4% of the variance in the median EC of pathways that include genes assigned based on all evidence (computational and experimental, EC = 0.14) and pathways that include genes assigned based on computational evidence (EC = 0.10). The small increase in co-expressed genes pairs when including experimental evidence is potentially due to the small fraction of genes that have experimental evidence (5,991 genes considering all evidence, 934 genes considering experimental evidence). Nonetheless, because annotation quality did have a measurable impact, only genes with experimental evidence were included in further analyses.

### Influence of the similarity measure used to assess EC

In addition to gene annotation quality, the similarity measure used to assess gene co-expression could impact pathway EC. Although PCC is among the most widely used similarity measures in co-expression studies, it does not deal with non-linear relationships as well as other similarity measures including Spearman’s rank correlation coefficient and mutual information (MI). Another consideration is that, all three similarity measures above consider only pairwise correlations, thus higher order correlations due to the influence of the other genes in the network are not considered. To assess the influence of higher order correlation, we also evaluated two approaches: (1) partial correlation, where the correlation between genes is calculated after controlling for the effects of other genes and (2) a graph model-based approach such as Bayesian Network (BN) where the strength of connection of a gene pair is determined by considering all genes in a network. To assess the impact of potential non-linearity and higher order correlations, we first calculated pathway ECs with seven different similarity measures including PCC, Spearman’s rank, two partial correlation methods (corpcor and partialcorr), adjusted and normalized MI, and transformed *p-*value of arc strength in a pathway BN (see Methods). To assess the statistical significance of EC values and control for false positive ECs, EC values were calculated with randomly chosen gene pairs for each pathway size and for each similarity measure. Thus, for each measure, a random EC distribution is available and used to determine the percentile value of a pathway EC (referred to as "EC percentile”). Thus, a high EC percentile indicates reduced probability that the observed pathway EC is spurious.

First we asked if the pathway EC percentiles are correlated among different measures (Fig 2B). For example, EC percentiles calculated with PCC were significantly positively correlated with the EC percentiles calculated with, in order of diminishing degrees of correlations, corpcor (PCC = 0.80, *p* = 2.35e-50), Spearman’s rank coefficient (PCC = 0.78, *p* = 1.67e-46), adjusted MI (PCC = 0.53, *p* = 5.23e-18), partialcorr (PCC = 0.39, *p* = 1.97e-09), BN (PCC = 0.30, *p* = 5.35e-06), and normalized MI (PCC = 0.17, *p* = 1.23e-02). Given the degrees of correlations in EC percentiles differ widely between measures, the similarity measures have significant impact on pathway ECs. Consistent with this notion, the number of pathways with ECs that are significantly higher than randomly expected (high EC pathways, >95^th^ percentile of the random EC distribution) vary widely depending on the similarity measure (Fig 2C). Among the measures, corpcor, PCC and Spearman’s rank allowed the highest numbers of high EC pathways to be identified. This finding is consistent with the finding of a recent study examining PCC, Spearman’s rank coefficient, MI, and other similarity measures [27]. Only five of the pathways have high ECs consistently regardless of similarity measures (Fig 2D). Importantly, consistent with the idea that non-linearity and higher order correlations can be important, the ECs of some pathways are only significant if a particular similarity measure is used (white boxes, Fig 2D). Notably, 17 and 10 pathways have high ECs only when the corpcor method and the BN-based measure were used, respectively (Fig 2D), illustrating the importance of higher order correlations. In addition, different methods of calculating partial correlations led to significant differences in high EC pathway recovery. As the corpcor method was optimized for genomic data analysis [50], our finding is justifiable that the results from corpcor is more informative than the results from partialcorr. For further analyses, we used PCC as the measure of gene co-expression as it is one of the most widely-used similarity measures and, along with Spearman’s rank and corpcor, uncover the highest numbers of high EC pathways.

### Influence of biological factors on pathway EC

**Fig 3:**
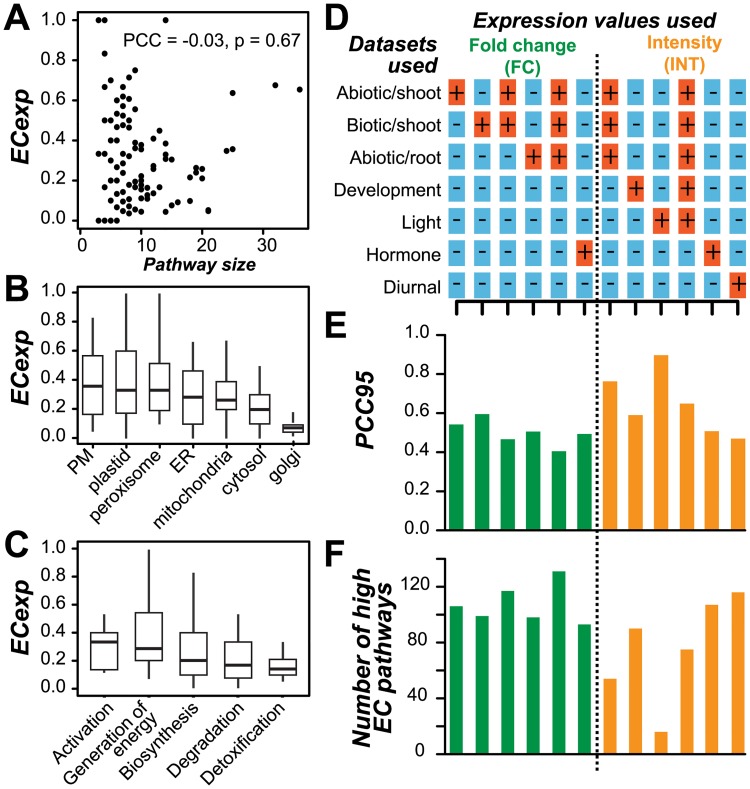
Impact of pathway size and other factors on EC. **(A)** Relationship between ECexp of a pathway and pathway size (the number of genes assigned to a pathway). **(B)** ECexp value distribution for pathway genes with products that have subcellular location annotations. PM: Plasma membrane. ER: Endoplasmic reticulum. **(C)** ECexp value distribution for different pathway classes (general pathway categories). **(D)** Datasets used to determine pathway ECs. A “**+**” indicates that the dataset in question was used (either individually or in combination) for the analyses depicted by bar graphs in (E) and (F). The columns in (D) correspond to those in (E) and (F). **(E)** The 95th percentile PCC values (PCC95) in the null distributions for each dataset or combination of datasets. PCC95 of combined datasets (stress fold change and light (L)+stress (S)+development (D) absolute intensity) are labeled in the bar plot **(F)** Number of pathways with high EC for each dataset and/or combination of datasets. Green: fold change values were used to calculate ECs. Orange: absolute intensity values were used for calculating ECs.

Next we explored biological factors that may influence pathway EC, including pathway size (the number of genes assigned to the pathway), subcellular location, pathway gene function, and evidence of co-regulation. We hypothesized that a pathway with a larger number of genes might have relatively more complicated modes of regulation beyond transcription, leading to low pathway ECs. In addition, gene products with similar functions tend to be co-localized and may be coordinately regulated [63], as is the case for photosynthesis and other chloroplast-related pathways [64]. However, pathway gene number was not significantly correlated with pathway EC (PCC = -0.03, *p* = 0.67; Fig 3A), and pathway gene product subcellular location was not associated with pathway EC (Fig 3B).

To assess whether the general biological functions of a pathway contribute to differences in EC between pathways, we compared ECs between five general pathway categories including activation, generation of precursor metabolites and energy, biosynthesis, degradation and detoxification. However, the significance of enrichment of these general categories was only marginal (Mann-Whitney Test, *p* = 0.05; Fig 3C). Interestingly, although the expression of gene pairs in the general category of generation of precursor metabolites and energy was not always significantly coherent, the specific pathways within—photosynthesis light reactions, chlorophyllide a biosynthesis I and aerobic respiration—had significantly higher ECs compared to random pathways (99^th^ percentile of pathway EC distribution). This finding suggests that EC, and more generally co-expression, is more relevant to more detailed levels of the functional classification hierarchy.

Transcriptional regulation is another major factor that could influence pathway EC. Genes that are co-regulated could have similar transcript profiles, and the differences in the degree of co-regulation may explain differences in pathway EC. To determine the extent of co-regulation, we asked how the presence of *cis-*regulatory elements differs among pathways. It is expected that pathway genes with similar sets of *cis*-elements in their promoters would have similar expression patterns and thus contribute to high pathway EC. We mapped 349 transcription factor binding motifs [36] to the promoters of all *A*. *thaliana* genes, and identified motifs that were over-represented in the promoters of pathway genes taking each pathway separately and comparing to all other genes. A total of 40 over-represented motifs were found for 17 pathways (S2 Table). However, there was no significant difference in EC between pathways with and without over-represented motif sites (Mann-Whitney Test, *p* = 0.66; S2B Fig). This was surprising given that the 349 motif dataset spans essentially all known *A*. *thaliana* transcription factor families, and transcription factors from the same family tend to have similar binding motifs [36]. Thus, the reason why high EC pathway genes do not necessarily have more shared motifs (S2B Fig) is not simply due to unknown transcription factor binding sites. This finding can also be due to complex interactions between binding sites, nucleosome positioning and other DNA properties [65]. We also evaluated post-transcriptional regulation by miRNA, but did not find a significant difference in EC between pathways with miRNA target genes and those that did not (Mann-Whitney Test, *p* = 0.31; S2C Fig). Given the dearth of genome-wide post-transcriptional and other levels of regulatory data in plants, it remains to be resolved if post-transcriptional regulation contributes to a lower pathway EC.

### Impact of datasets used to evaluate pathway EC

Among the factors studied—the size of the pathway, subcellular location, functions of pathway genes, and evidence of shared transcription factor binding sites—none significantly impact pathway EC. We next asked whether the expression dataset has a major impact on whether the EC for a pathway is high or low. The analyses described so far were performed using an environmental stress dataset consisting of 112 experiments including biotic and abiotic stress treatments in shoot and root [41]. Low pathway EC values could reflect the fact that pathways are only relevant to one type of stress (biotic or abiotic) and a large compiled dataset fails to capture the underlying patterns of co-expression. To address this possibility, we first calculated the random gene pair correlations for three subsets of the environmental stress gene expression dataset: shoot abiotic, shoot biotic, and root abiotic. PCC95 values were higher for subsets (PCC95 = 0.51–0.60) of the stress dataset than for the entire dataset (PCC95 = 0.41; Fig 3D and 3E), indicating that the difference in gene expression between experiments within a dataset, i.e. data heterogeneity, was lower when the samples were divided into biologically relevant subsets. Consistent with this, the average sample correlation within each of the shoot biotic, shoot abiotic, and root abiotic subsets is higher (0.46, 0.15, and 0.19 respectively) than the entire environmental stress dataset (0.13, Mann-Whitney Test using all pairwise sample PCCs, *p* = 8.73e-26, 2.10e-05, 1.93e-144 respectively). Due to the impact of data heterogeneity, fewer high EC pathways tend to be recovered from individual stress datasets compared to combined datasets (Fig 3F).

To test whether these findings are specific to the environmental stress data, an additional four expression datasets were analyzed (development, light, hormone, and diurnal; Fig 3D; S1B Table). We found that the threshold PCC95 values of these datasets were significantly negatively correlated with the number of high EC pathways (PCC = 0.97, *p* = 1.10e-08). Thus, because PCC95 is negatively correlated with data heterogeneity (as discussed in the previous section), higher data heterogeneity likely allows more co-expressed pathway genes to be recovered. Data heterogeneity can be influenced by which datasets are combined and how the expression data are processed and transformed. Combining datasets tends to increase data heterogeneity and thus leads to a better recovery of pathway genes based on co-expression (Fig 3F). Dataset processing also has an effect on data heterogeneity. For example, datasets that were processed to obtain fold change values had a substantially lower PCC95 (median PCC95 of fold change datasets = 0.41; Fig 3E) than that of the absolute intensity dataset (median PCC95 of intensity datasets = 0.76; Fig 3E), although this was not true for the hormone dataset (Fig 3E). Taken together, these results reveal that dataset transformation approaches and nature of the expression dataset impact the threshold for defining significant co-expression and thus significantly shapes pathway EC.

### Influence of individual vs. combined stress datasets on pathway EC

**Fig 4:**
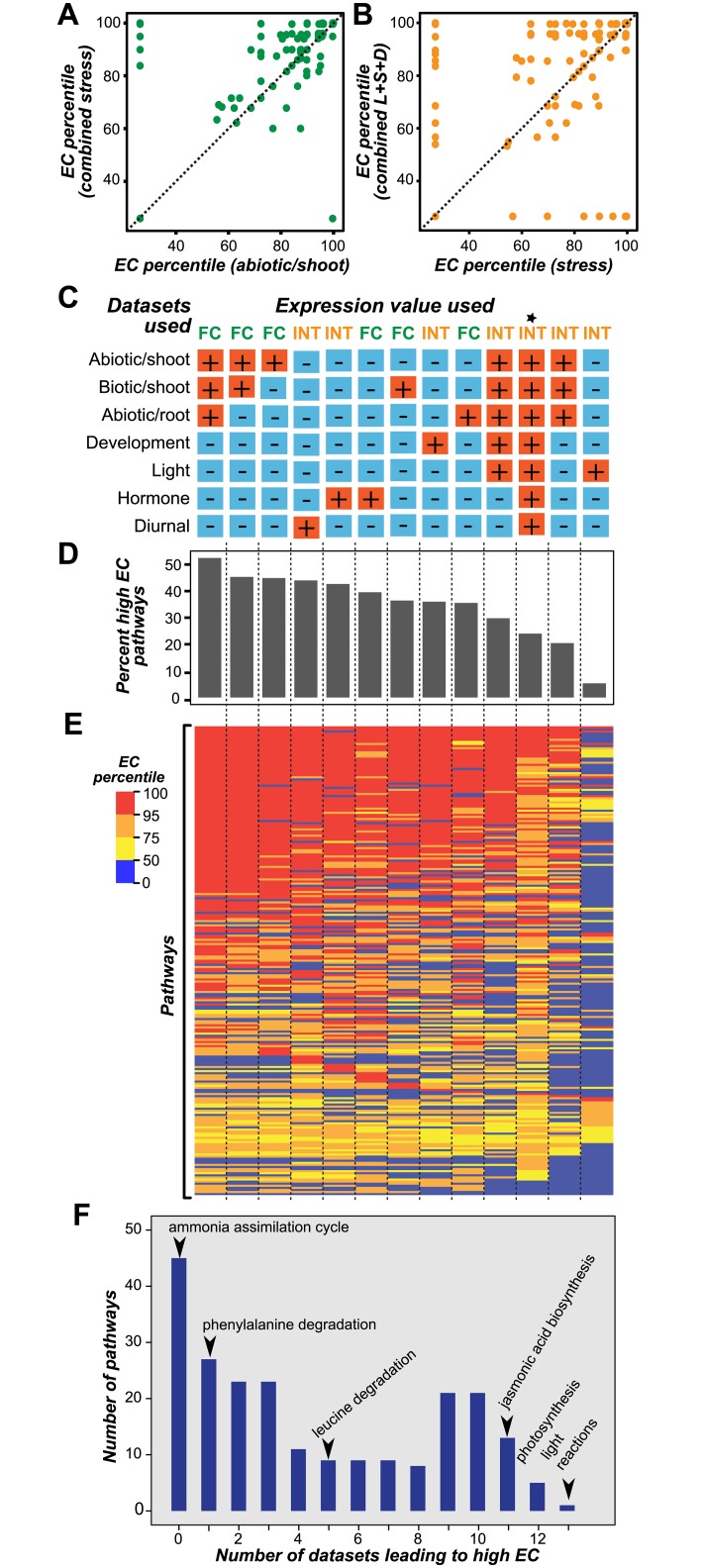
Impact of datasets on pathway EC percentile. **(A)** Relationship between pathway EC percentiles calculated using the combined stress gene expression dataset and those calculated based on one of the individual stress datasets, abiotic/shoot. **(B)** Relationship between pathway EC percentiles calculated using the light, development and stress combined dataset and those calculated based on individual dataset, stress. In (A) and (B) the dashed line represents *y* = *x*, and each dot represents a pathway. **(C)** Individual and combinations of datasets used to determine pathway EC Percentiles. *: NASCArray consisting of all the datasets listed here as well as additional datasets (~700 samples). The columns in (C) correspond to those in (D) and (E). **(D)** Bar plot of percent high EC pathways using different expression datasets **(E)** Heat map of pathway EC percentiles from 13 gene expression datasets. Dark red: EC percentiles≥ 95. Orange: 95 > EC percentiles < 75. Yellow: 75 > EC percentiles <50, Blue: 50 > EC percentiles < 0 **(F)** Histogram of the numbers of datasets leading to high EC values for each pathway. Example pathways are labeled with an arrow.

A wide range (5%-53%) of pathways have significantly high ECs depending on the dataset used (Fig 3F). This pattern led us to question whether some datasets are more informative than others in recovering specific pathways. To assess this, pathway EC percentiles were calculated for each dataset separately (S3A Fig). Note that for each expression dataset analyzed, we picked half a million pairs of randomly chosen genes from a total of ~22,000 to establish background correlations and selected the correlation threshold at the 95th percentile of the random correlation distribution. Because dataset heterogeneity influenced the threshold values used to determine gene co-expression (Fig 3E), we first asked whether larger, combined stress datasets were more informative (i.e. had higher pathway EC percentiles) compared to smaller, individual datasets (Fig 4A; S3B and S3C Fig). The combined stress dataset had a higher median EC percentile (95.6) compared to the individual datasets (89.5–89.9) (S1C Table). For example, the monoterpene biosynthetic pathway had an EC percentile of 99.6 based on the combined stress dataset, but the values ranged from 26.3 to 89.9 for individual datasets (S1C Table).

By contrast, in >14% of the pathways, the EC percentiles determined with the individual datasets were higher than those based on the combined dataset (Fig 4A; S3B and S3C Fig). For example, the lipid dependent phytate biosynthesis I pathway had an EC percentile of 99.5 when the root abiotic stress dataset was used compared with EC percentiles<27 for all other individual and combined datasets (S1C Table). Another example is the cuticular wax biosynthetic pathway, which had an EC percentile of 99.7 calculated from the shoot abiotic stress data, but had EC percentiles of 26.4 and 26.6 when root abiotic and shoot biotic stress datasets were used, respectively (S1C Table). This is consistent with the role of cuticular wax in protecting the shoot from drought and other stresses [66,67] and the co-regulation of its biosynthetic genes [68]. Similarly, indole-3-acetic acid (IAA) degradation genes have EC percentiles of 99.9 and 26.3 using root and shoot abiotic stress datasets, respectively (S1C Table), consistent with the finding that IAA degradation products have been mainly detected in roots [69,70].

These findings lead to the conclusion that EC among genes in the same pathway is strongly influenced by whether individual or combined stress datasets are used, particularly if the pathway in question is biologically relevant to the experimental conditions of the dataset. Thus, it is important to test multiple individual and combined datasets for finding the optimal EC for a pathway. It should be noted that, the small numbers of samples in individual datasets mean less power in detecting co-expression; however, we were able to recover high EC pathways with individual datasets. This is because we have included randomized background information for different sized datasets in calculating threshold pairwise similarities for determining EC and in calculating threshold EC values for identifying pathways with significantly high ECs. A smaller dataset where spurious correlations are expected will have a correspondingly higher threshold because the correlations between randomized gene pairs will be higher.

### Robustness in recovering pathway genes when using different datasets

To determine whether the conclusion that EC is strongly influenced by stress (S) datasets is generalizable to non-stress ones, we further increased the dataset size by including light (L) and developmental series (D). We found that when using dataset L, S, D, and combined (L+S+D) datasets, 12, 46, 81, and 96 pathways had significantly higher than expected EC, respectively. Although the combined dataset was the best for uncovering more pathways, the EC percentiles were higher for some pathways when individual datasets were used (Fig 4B; S3D and S3E Fig). Two interesting examples are the *trans*-zeatin biosynthesis and the iron reduction/absorption pathways. These pathways only had significantly high EC when using the light dataset (S1C Table). Fluctuations in light conditions can alter the expression of *trans*-zeatin biosynthesis genes [71]. In addition, iron is a central component of chlorophyll. One iron reduction gene, *FRO6*, contains multiple light-responsive elements, and another, iron reduction gene *FRO7*, has an expression pattern similar to *FRO6* [72,73]. Consistent with our discussion on the impact of individual and combined datasets in the previous section, these findings indicate that dataset choice impacts the optimal recovery of pathway genes. Next, we asked how data transformation impacts pathway EC percentile. The EC percentiles determined from fold change and absolute intensity were significantly positively correlated for the stress (PCC = 0.38, *p* = 4.90e-9) and hormone (PCC = 0.57, *p* = 1.17e-20; S3F and S3G Fig) datasets. Despite these significant correlations, data transformation still resulted in a >50 percentile difference in EC for 27% and 12% of pathways using stress and hormone datasets respectively (S1C Table).

Based on our results, it is important to test datasets according to the pathway of interest, but do more expression data samples necessarily lead to better pathway recovery? To answer this question, we compared pathway EC percentiles across 12 individual and combined datasets (Fig 4C). We found that the stress dataset yielded the highest percentage of high EC pathways (53%) among larger, combined datasets analyzed (Fig 4C). To further assess whether using a much more inclusive, more conditionally independent dataset compared to the 12 datasets we used, would increase the recovery rate of high EC pathways, we analyzed NASCArrays dataset with >700 samples [44]. We found that 24% of the pathways had high EC with the NASCArray dataset. This recovery rate was lower compared to a much smaller dataset such as the stress set, where 53% of pathways had high ECs (Fig 4D). Thus more is not necessarily better. This is because the overlap in within and between pathway expression correlations was larger when the NASCArray dataset was used compared to the stress dataset (S4A and S4B Fig), indicating that it was harder to distinguish within and between pathway gene pairs using the NASCArray data.

Next we asked if some pathways have significantly high EC regardless of the dataset used (i.e. are robust). Among pathways, 180 had significantly high EC in >1 datasets (Fig 4E), but photosynthesis light reactions was the only pathway that had significantly high EC in all datasets. This is consistent with earlier findings that light reaction genes are tightly co-regulated [74]. In addition to photosynthesis light reactions, jasmonic acid biosynthesis, aliphatic glucosinolate biosynthesis side chain elongation cycle, fatty acid elongation, palmitate biosynthesis II and chlorophyll a degradation II were also among the most robust pathways in terms of EC.

On the other end of the spectrum, 15% of the 179 pathways with significant EC had significantly high EC in only one dataset (e.g. phenylalanine degradation; Fig 4F), further indicating the importance of dataset selection for co-expression associations with unknown genes. In addition, 21% of the pathways (e.g. ammonia assimilation cycle; Fig 4F) did not have significant EC regardless of the dataset used; indicating that additional datasets may be required and/or these pathways are mainly regulated at levels beyond transcription. Given that many pathways had significant EC when a particular dataset was used, we asked how many individual datasets are required to recover the 180 pathways with significant ECs. Interestingly, when datasets are included one at a time, the number of pathways with significantly high EC initially increased but appeared to be saturated after the addition of 11 datasets (S3H Fig).

Taken together, although genes within pathways can have similar expression patterns, this similarity is best recovered after experimenting with a number of different individual and combinations of datasets as well as with data transformations. In addition, although data heterogeneity increases the number of pathway genes that can be recovered, combining datasets is not necessarily the best approach for all pathways. Comparing to 5–53% high EC pathways that can be discovered when datasets are used individually, combining the analysis results of the individual datasets led to the finding that 80% pathways have high ECs.

### Clusters as predictive units of pathways

**Fig 5:**
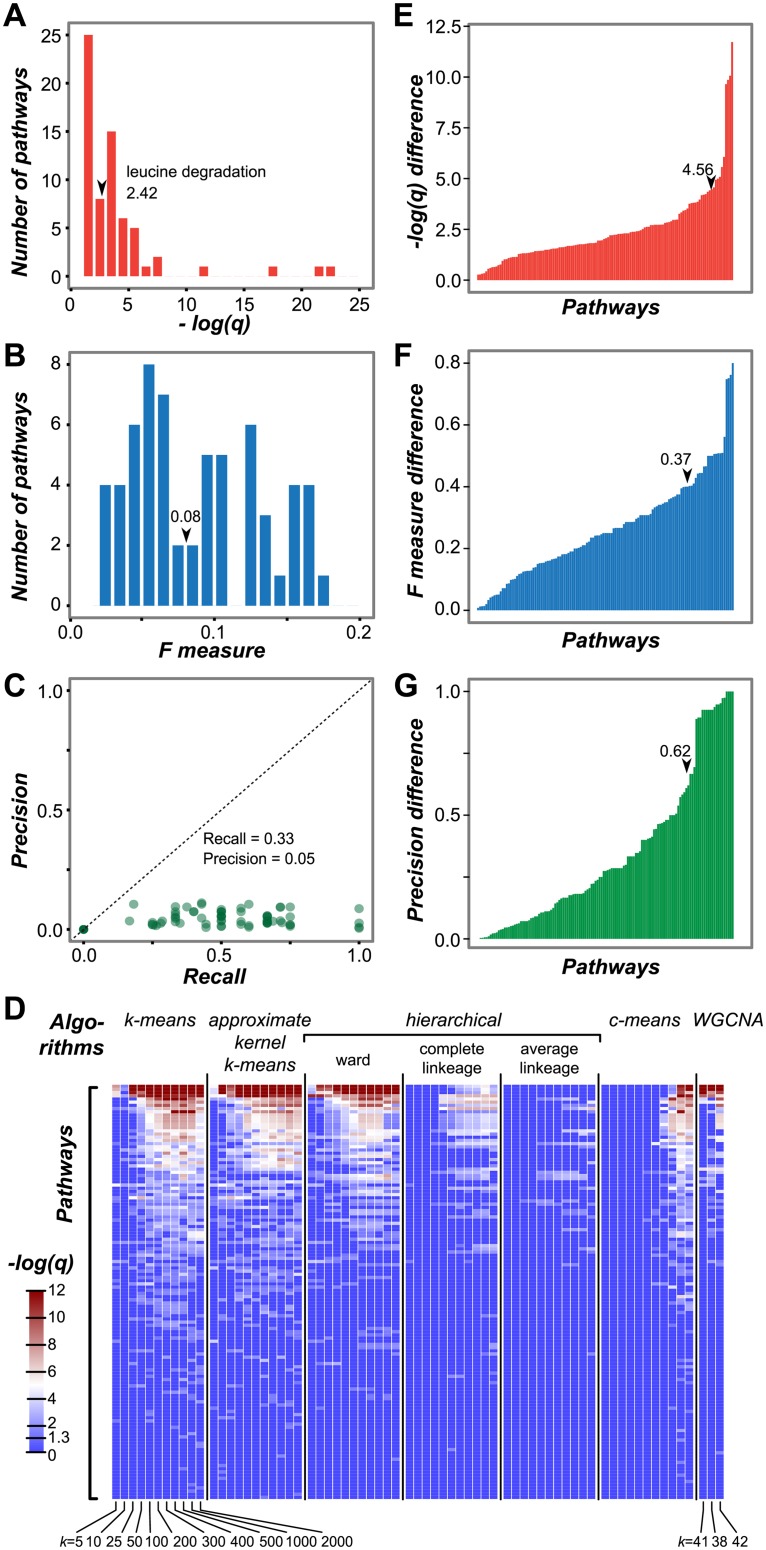
Performance of clusters in predicting pathways. **(A)** Histogram of the maximum scores (-log(*q*)) for over-representation of pathways within clusters. **(B)** Histogram of the maximum F measures for prediction of pathway membership based on cluster membership. **(C)** Relationship between precision and recall for clusters. In (A-C), clusters were generated using *k*-means with *k* = 100. **(D)** Heat map of over-representation scores obtained from different individual and combined clustering algorithms (top) and cluster numbers (bottom) Color represents over-representation scores (-log(*q*)) from 0 to 12. Scores less than 1.3 are indicated by dark blue. Scores more than 1.3 are represented by a spectrum of light blue to red. Pathways in the heat map are sorted based on the number of times that they are over-represented in the clusters, high to low. **(E)** Bar plot showing the difference between overall maximum over-representation score—the highest score from any single cluster—and the over-representation score from clusters generated using *k*-means, *k* = 100 for each pathway. **(F)** Bar plot showing the difference between the overall maximum F measure—the highest score from any single cluster—and the F measure from clusters generated using *k*-means, *k* = 100 for each pathway. **(G)** Bar plot showing the difference between maximum Precision—the highest score from any single cluster—and the Precision from clusters generated using *k*-means, *k* = 100 from each pathway. Arrow: performance values for the leucine degradation pathway.

Clustering genes based on similar expression profiles is commonly performed to find genes that are functionally related [75]. In the best-case scenario, most of the genes in a pathway would be in the same cluster, and the remaining genes in the cluster could be tested to see if they have functions similar to the pathway genes. To evaluate the extent clustering would give us this scenario, we first employed one of the most widely used clustering algorithms, *k*-means, to group ~22,000 genes in the stress gene expression dataset. To determine the optimal *k*, there are multiple proposed statistical methods including Bayesian Information Criterion (BIC) [76], gap statistic based on the elbow plot [77], and silhouette score [78]. Although these measures have been successfully implemented in simulated datasets where the grouping is apparent [79], there is no best method in determining the number of natural groups of the high-throughput genomics data and often researchers have to try multiple *k* values [80,81]. In our initial analysis, we used elbow plot to define *k*. We computed within cluster sum-of squares for a range of *k* values starting from 5 clusters and going up to 2000 (S5A Fig). Even though there was no clear elbow point, the decrease in the within sum of squares was apparent when *k* = 100 which was used for *k-*means clustering. Once the 100 clusters were obtained, over-representation analysis was used to assess how well pathway and cluster membership coincide and an over-representation score was defined (see Methods). Clusters with significant over-representation scores (*q* <0.05) were analyzed further (Fig 5). Our expectation for an ideal clustering result was a low *q*-value (~0). Only 30% of the pathways were found to be over-represented in >1 cluster, and 38% pathways had an over-representation score < 2 (0.01 < *q* < 0.05).

As significance alone does not tell us to what extent each cluster is informative in finding additional genes associated with the pathway of interest, we evaluated each clustering result as a prediction problem, where a gene’s membership in a cluster is used to predict its membership in a particular pathway. The performance of the clustering results was evaluated using the F measure, which is the harmonic mean of Precision and Recall. Here precision is the proportion of the number of genes that overlap between a cluster and a pathway to the number of genes in the cluster. Recall is the proportion of the number of genes that overlap between a pathway and a cluster to the number of genes in the pathway. F measures can range from 0 and 1 and higher F measures suggest that both Precision and Recall are high. Precision, Recall and F measures were calculated for every pathway-cluster combination when there was a significant enrichment (*q* <0.05; Fig 5B and 5C). We expected high Precision (~1) for the most informative clusters, but the highest precision among cluster-pathway combinations was 0.11. In one cluster, 11% of the genes belong to the “glucosinolate biosynthesis from the tryptophan pathway”. The same cluster also yielded the highest F measure (0.18). This result suggests that there is a need to improve this clustering result, potentially by using different clustering algorithms and parameters. This is explored further in the next section.

### Impact of clustering algorithms and parameters on the identification of pathway genes

In the analyses described so far, we used only one clustering algorithm (*k*-means) and fixed parameters (Euclidean distance, *k* = 100). Next we assessed how additional clustering algorithms and clustering parameters (number of clusters defined, distance measure, and number of runs) impact the identification of co-expressed gene clusters and how this in turn impacts the identification of genes with similar functions. Five algorithms were applied to the stress expression dataset using different parameters including number of clusters (*k*), consistency among runs, and other algorithm-specific parameters, to obtain 366 different clustering results (S3 Table). Although some of the algorithms (*k*-means, approximate kernel *k*-means, *c*-means) often yield local optima instead of an overall best result, clustering runs with the same algorithm and parameters gave very similar results (average PCC among 10 runs = 0.8–1.0). Therefore, only the maximum over-representation score from 10 runs is shown (Fig 5D).

We found that the choice of *k* is important; regardless of the algorithm, smaller *k* values resulted in low over-representation scores (Fig 5D) and a smaller number of pathways over-represented among clusters (S5B Fig). This is likely due to the fact that smaller *k* values lead to larger sized clusters that contain genes from multiple pathways. We also found that the number of members in a cluster that overlap with members of a pathway differs depending on the algorithm used; *k*-means was the best performing algorithm, followed by approximate kernel *k*-means and hierarchical clustering with the Ward algorithm (S5B Fig). Overall, with all clustering methods combined, we were able to recover 131 pathways out of 225 (64 more pathways than when only *k*-means, *k* = 100 was used). In contrast, 95 out of 225 pathways were not over represented in any of the clusters, and 22 pathways were only over-represented in one algorithm-parameter combination (S5C Fig). Taken together, the clustering approach is not deterministic; the parameters used influence co-expression associations. Therefore, it is important to evaluate multiple algorithms and parameters to recover pathways of interest.

Multiple algorithm-parameter combinations were examined (e.g. an example combination: *k*-means, *k* = 100), to quantitatively assess the degree of improvement in performance measures. First, clusters from 69 algorithm-parameter combinations were generated (Fig 5D). For each pathway, we asked what the maximum over-representation score was among the clusters from all combinations. This maximum score was then compared to the over-representation score of clustering results from our standard method discussed above (*k*-means, *k* = 100; Fig 5E). We found that the over-representation scores of the best clusters were increased by an average of 1.40 (25-fold better *q*-value) compared to the score when only one algorithm/parameter was used. We also evaluated clustering performance using F measure (improved by an average of 0.15; Fig 5F) and Precision (improved by an average of 0.20; Fig 5G). These results reinforce the importance of considering multiple algorithms and parameters to maximize pathway-cluster overlap. Furthermore, for algorithms requiring a predefined *k*, the *k* value may be different depending on the pathway one would like to recover and it is necessary to try out multiple values for the best results. Thus, selecting a presumably optimal *k* may yield a more natural grouping of the entire dataset but at the expense of uncovering clusters representing individual pathways.

We should emphasize that, although considering multiple clustering parameters allow recovery of 93 pathways, there are still 96 pathways that were not recovered by the five algorithms used in this study (S6 Fig). This may be because genes in these pathways do not have highly coordinated expression patterns and have low pathway ECs. Consistent with this interpretation, more high EC pathways tend to be recovered by clustering compared to low EC ones (Fishers exact test, *p* = 4.56E-12; S6 Fig). We should also emphasize that the scores used to assess the clustering performance ignore the possibility that some genes in the clusters will be novel pathway components. The presence of these genes reduces the over-representation score, precision, and F-measure. These novel pathway component genes are prime candidates for further functional characterization using genetic or biochemical analysis.

### Using leucine degradation gene phenomics data to validate co-expression associations

We established that the degree of gene co-expression in some pathways is influenced by dataset and data transformation and that it is important to use multiple algorithms and parameters when identifying clusters based on co-expression. To demonstrate that novel pathway components can in fact be recovered as a result, we used phenomics data to validate novel gene components of the leucine degradation pathway [57,58]. We chose to focus on leucine degradation because it is among the most over-represented pathways in co-expression clusters (Fig 5), and many components of the leucine degradation network remain to be discovered in plants [82,83]. Eighteen novel genes that were not annotated to leucine degradation in the AraCyc database are consistently found in clusters (≥10 clustering results; S4 Table) that are over-represented with 12 annotated leucine degradation genes. Among these genes, AT1G55510, a branched-chain alpha-keto acid decarboxylase E1 beta subunit, was recently shown to be involved in leucine degradation [82] but has not yet been annotated as such. The fact that AT1G55510 is consistently found in the same clusters as leucine pathway genes prompted us to examine the rest of the genes that cluster with leucine degradation genes (S4 Table) for involvement in leucine degradation.

**Fig 6:**
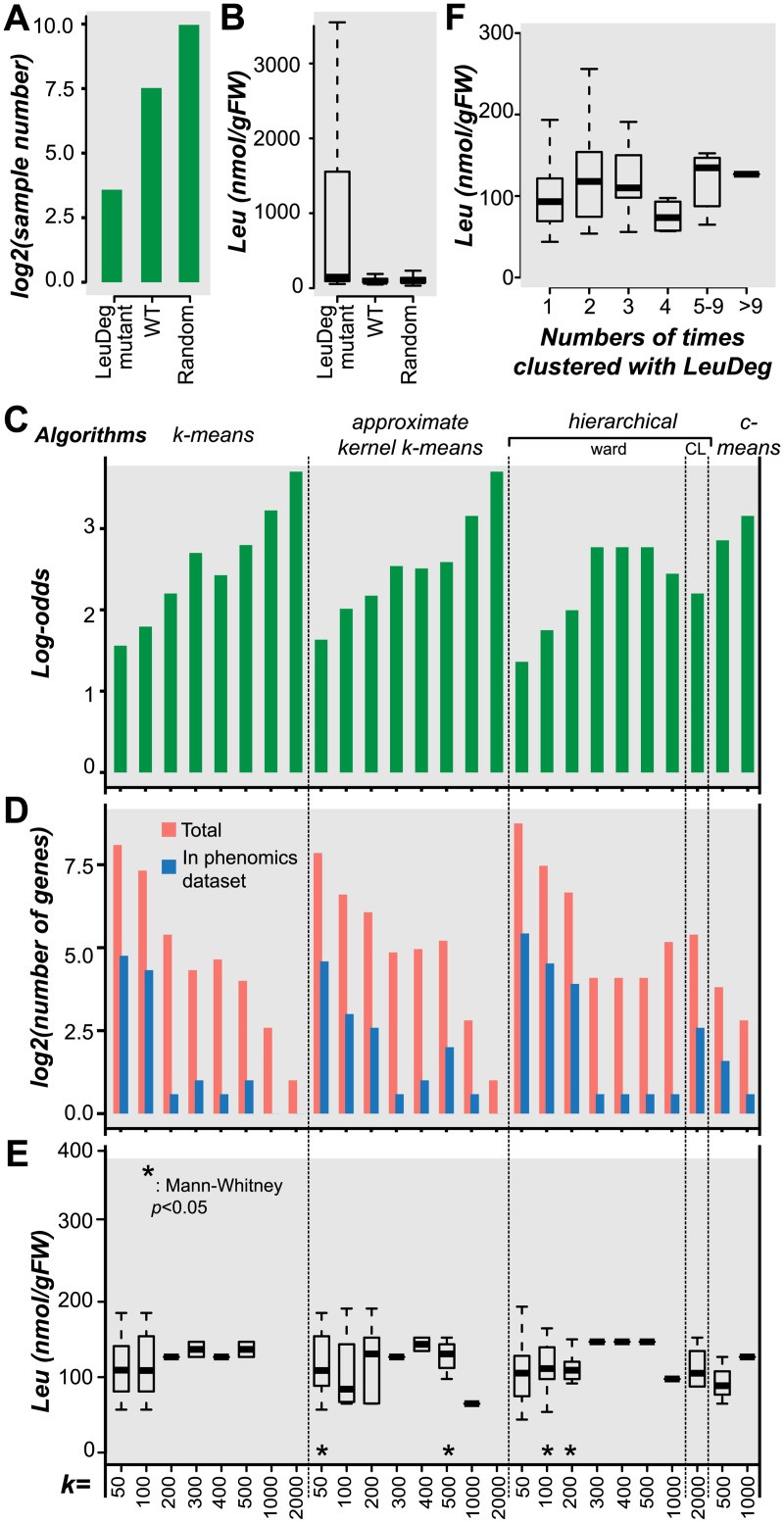
Assessing the validity of co-expression associations with leucine measurement data. **(A)** Log_2_ of the number leucine degradation (LeuDeg) gene mutants, random gene mutants, and wild-type (WT) control plants that were included in the analysis of leucine levels. **(B)** The absolute leucine levels (nM/gFW) in the same three types of genetic background as in (A). **(C)** Log-odds values (log ratio between the proportion of leucine degradation genes in a cluster to the proportion of non-leucine degradation genes in the same cluster) of clusters that are enriched in leucine degradation genes identified using different algorithm-size parameter combinations. **(D)** Log2 of the number of genes that cluster with leucine degradation pathway genes (over-representation score >1.3) for each algorithm-size parameter combination. **(E)** Box plot showing the absolute seed leucine levels (nM/gFW) in plants with T-DNA insertions in genes clustered with leucine degradation pathway genes (enrichment score >1.3) for each algorithm-cluster size parameter combination. *: the groups of genes where mutant leucine levels are significantly greater than in wild type. **(F)** The absolute seed leucine levels (nM/gFW) of T-DNA insertion mutants of genes that cluster with leucine degradation genes. Binning in x-axis depends on the number of times that each gene clusters with leucine degradation genes considering all clustering results.

We hypothesized that previously unknown associations deduced from co-expression clusters could be verified based on their mutant phenotype data. To test this hypothesis, we used a published phenomics dataset that includes free seed leucine levels for mutants in more than 5,000 genes (Fig 6A) [58]. The free leucine levels (nmol/g fresh weight) of leucine degradation gene mutants are expected to be more similar to genes within the same cluster than to wild type plants or randomly chosen mutants. As expected, the leucine degradation enzyme genes had higher leucine levels than mutants in random genes and wild-type plants (*p* = 0.05 and 0.04 respectively; Fig 6B). Next we evaluated the clusters that were over-represented with leucine degradation genes by calculating the log ratio between the proportion of leucine degradation genes in a cluster to the proportion of non-leucine degradation genes in the same cluster. Note that, as *k* increases, the log ratio tends to increase (Fig 6C). This trend is potentially due to increased statistical power to identify over-representation in smaller sized clusters. Among these clusters, hierarchical clustering with the Ward algorithm (*k* = 100 and *k* = 200) and approximate kernel *k*-means (*k* = 50, *k* = 400 and *k* = 500) yielded clusters that had genes (Fig 6D) whose leucine levels were significantly higher than the wild-type measurements (*p* = 0.01–0.05; Fig 6E; S5 Table). Thus, some genes in those co-expressed clusters are likely involved in leucine degradation. Nonetheless, the differences in leucine levels between mutants of genes in the cluster and wild-type plants were small (Fig 6E). This may be due to the fact that some co-expressed genes are false positives. However, some known leucine degradation pathway gene mutants also do not have dramatic differences in leucine level compared to wild-type (Fig 6B) and this may also explain the small effect size. We next asked whether a gene that consistently clusters with leucine degradation genes—regardless of the algorithm and parameters used—tends to be a better pathway gene candidate than one that does not. Mutants in genes that were retrieved from three separate clustering results had significantly higher leucine levels than mutants in random genes and wild-type plants (*p* = 0.03 and 2.60e-3 respectively; Fig 6F), indicating that consistency may serve as a criterion to increase confidence in candidate genes.

## Discussion

A large number of high-throughput omics data are accumulating. Of these, transcriptome data are the most abundant, covering multiple tissues and conditions, and have been widely used to generate hypotheses about gene functions. Since almost the first microarray studies, researchers have used the guilt-by-association approach to make useful predictions about gene functions. This approach is based on the hypothesis that genes encoding proteins of shared function are more likely to have common features such as gene expression patterns. Here we show that even though this approach is useful, there are many limitations to co-expression-based functional inferences and that these limitations can be potentially overcome through methodological considerations that include pathway gene annotation quality, expression dataset used, clustering algorithms, and the use of an independent dataset such as the mutant phenotype data used here to maximize the utility of co-expression relationships in hypothesizing gene functional relations.

By evaluating within-pathway gene expression correlation based on the EC measure, we show that genes encoding proteins involved in the same pathway do not necessarily co-express. For example, only 5% of pathways have significantly high EC using a light treatment dataset. For the remaining 95% of pathways, pathway genes may not be coordinately expressed and/or the light dataset is not informative. For some pathways, co-expression will be ill-suited due to gene sharing among pathways (thus multiple mode of regulation), requirement for condition-specific expression data that are not available, and/or that coordinated regulation of the pathway is at a level beyond transcription. In other situations, several approaches could be taken to improve the recovery of pathways with high EC. By filtering genes based on annotation, it might be possible to obtain a core set of genes that are co-expressed. In addition, using expression datasets of different type (e.g. treatment and/or tissue types), complexity (e.g. individual or combined), and transformation method (e.g. fold change or absolute intensity value) could be effective.

In this study, we have demonstrated that clustering algorithms and parameters impact the ability to find novel pathway genes. Thus, by relying on a single algorithm and a single parameter—as is most commonly done in published studies—co-expression associations with functional implications might be missed. For any pathway being analyzed it is necessary to find the optimal algorithm and parameters to identify clusters that contain the majority of the known pathway genes. We also demonstrated that using one particular clustering algorithm-parameter combination, in most cases, does not lead to clusters that have optimal overlaps in gene memberships with pathways. Instead, for the best result, we need to consider multiple algorithms and parameters. The methodological considerations we had in this study reflect the multi-parameter nature of co-expression based analyses. Studies that include co-expression based approaches should involve rigorous testing of multiple variables ranging from the pathway of interest to expression dataset and clustering algorithm.

## Supporting Information

S1 Fig Boxplots of EC values distributions of overall and selected GO-BPs and Aracyc pathways. The order of x-axis is based on the median EC.
(PDF)

S2 Fig Factors that potentially influence pathway EC. **(A)** Relationship between the proportion of genes with experimental evidence and EC. EC is shown on the *x*-axis. **(B)** ECexp distribution of pathways with and without enriched motifs. **(C)** ECexp distribution of pathways that have miRNA target genes and of pathways those do not.
(PDF)

S3 Fig Randomized pathway EC distributions and EC percentiles from multiple datasets. **(A)** Left panel: Individual and combinations of datasets used to determine pathway EC percentiles. A “+” indicates that the dataset in question was used (either individually or in combination) for the distributions depicted in the Right panel. Right panel: Distribution of randomized pathway EC. Pathway gene membership was randomized 100 times. Median EC per pathway size is shown in this distribution. **(B)** Relationship between pathway EC percentiles calculated using the combined stress gene expression dataset and those calculated based on one of the individual stress datasets, biotic/shoot. **(C)** abiotic/root. **(D)** Relationship between pathway EC percentiles calculated using the light, development and stress combined dataset and those calculated based on individual dataset, development. **(E)** light. **(F)** Relationship between pathway EC percentiles calculated using the fold change and absolute intensities for the stress gene expression dataset. **(G)** Relationship between pathway EC percentiles calculated using fold change and absolute intensities for the hormone gene expression dataset. Dashed line: *y* = *x*. Each dot represents a pathway. **(H)** The change in the number of pathways with high EC (*y*-axis) with the addition of more expression datasets (*x*-axis).
(PDF)

S4 Fig Distinguishing gene pairs within and between pathways using a condition-dependent dataset and a condition-independent dataset. **(A**) Distributions of PCC values of within pathway gene pairs (light red) and between pathway gene pairs (light blue) using the condition-dependent, stress dataset. Red line: PCC at the 95^th^ percentile of between pathway gene pair PCC distribution. **(B)** Same as (A) but using the condition-independent, NASCArray dataset.
(PDF)

S5 Fig The extent to which metabolic pathways are over-represented in co-expression clusters. **(A)** Elbow plot showing within cluster sum of squares for *k* = 5–2000. **(B)** The number of pathways over-represented (y-axis) in clusters obtained using different algorithms and cluster numbers (x-axis). **(C)** Distribution of the number of times that a pathway is over-represented in a cluster (sorted high to low).
(PDF)

S6 Fig The extent to which high EC pathways are over-represented in clusters. The relationship between the pathway-cluster over-representation score (y-axis) and pathway EC percentile (x-axis). Horizontal red line: over-representation score = 1.3, corresponding to adjusted *p*-value of 0.05. A pathway is considered over-represented if the over-representation score is >1.3. Vertical red line: EC percentile = 95. High EC pathway has an EC percentile>95. The insert is the contingency table for testing (Fisher’s exact test) whether the number of high EC pathways is higher among over-represented clusters than randomly expected.
(PDF)

S1 Table Pathways and expression datasets used. **(A)** Pathway ECs/Random pathway ECs using stress expression datasets. **(B)** Expression datasets used in this study. **(C)** Pathway EC Percentile matrix.
(XLSX)

S2 Table Presence of potential regulatory sequences in pathway gene promoters. (XLSX)

S3 Table Co-expression clustering results. **(A)** Clustering algorithms used **(B)** Over-representation scores of pathways from 10 runs of *k*-means, approximate *k*-means and *c*-means. **(C)** Over-representation scores of pathways considering all algorithms.
(XLSX)

S4 Table List of genes that cluster with leucine degradation pathway genes. (XLSX)

S5 Table Statistics for comparisons of leucine levels between samples. (XLSX)
