# Supplementary figures and images for: Utility and Limitations of Using Gene Expression Data to Identify Functional Associations
Source: PLoS Comput Biol. 2016 Dec 9;12(12):e1005244. doi: 10.1371/journal.pcbi.1005244 (PMC5147789; doi:10.1371/journal.pcbi.1005244)

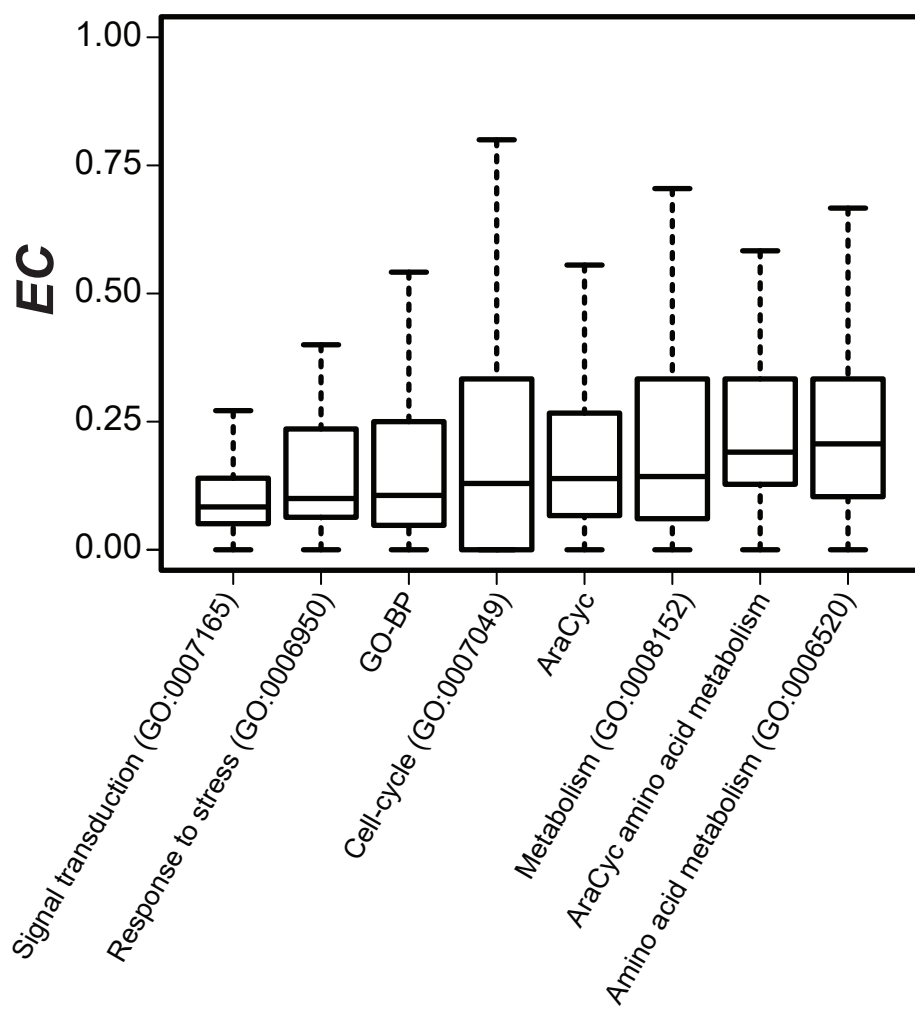

Supplement: S1 Fig — The order of x-axis is based on the median EC. (PDF) [file pcbi.1005244.s001.pdf]

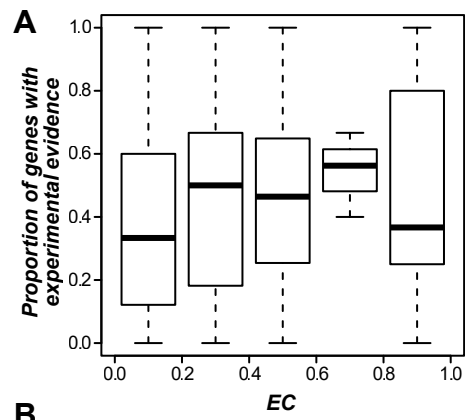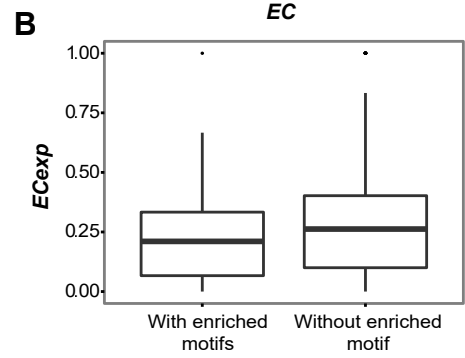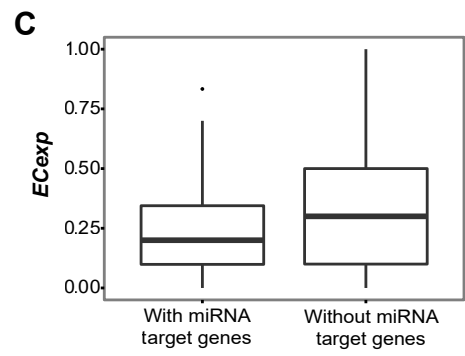

Supplement: S2 Fig — (A) Relationship between the proportion of genes with experimental evidence and EC. EC is shown on the x-axis. (B) ECexp distribution of pathways with and without enriched motifs. (C) ECexp distribution of pathways that have miRNA target genes and of pathways those do not. (PDF) [file pcbi.1005244.s002.pdf]

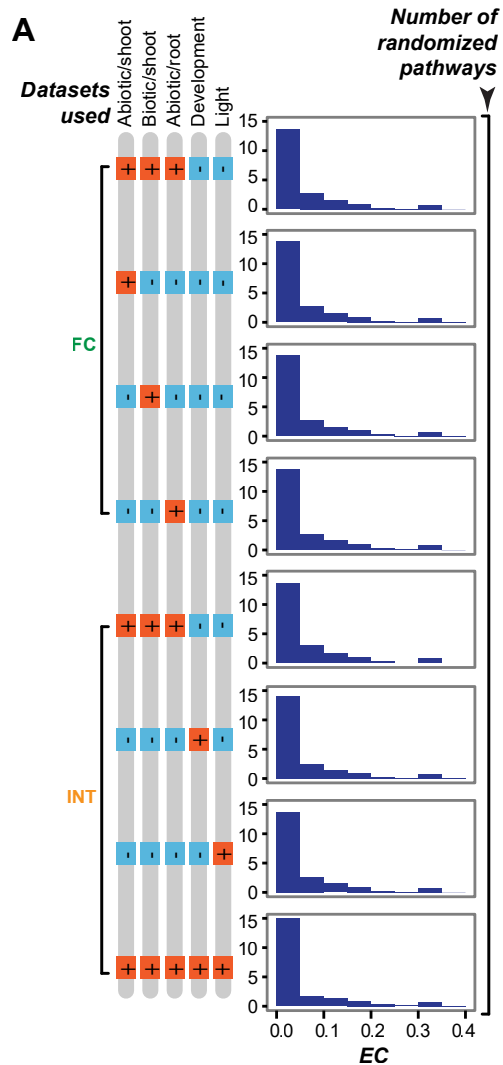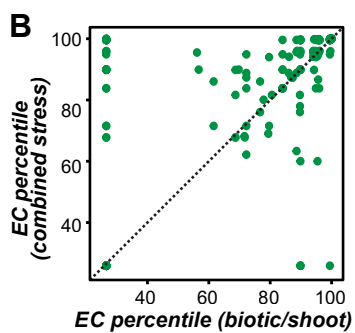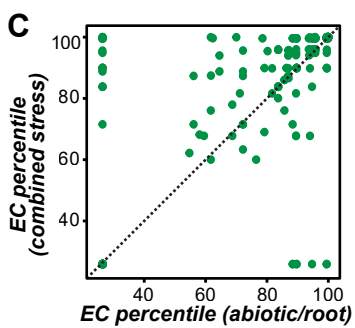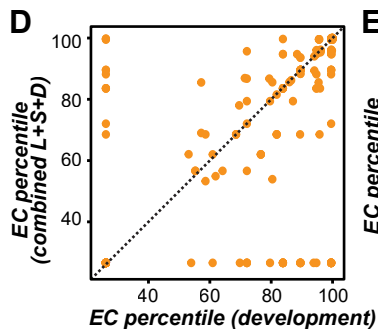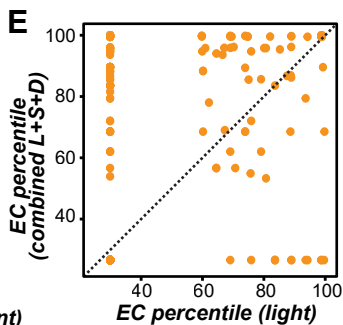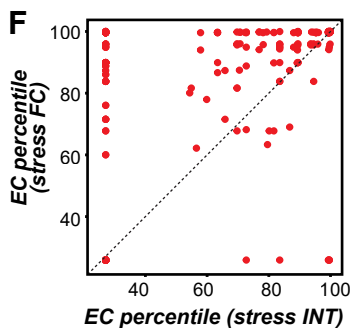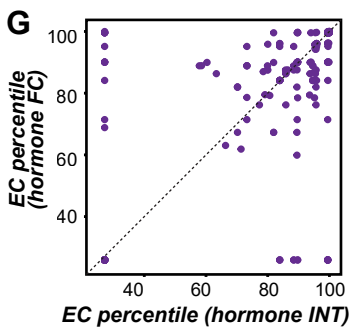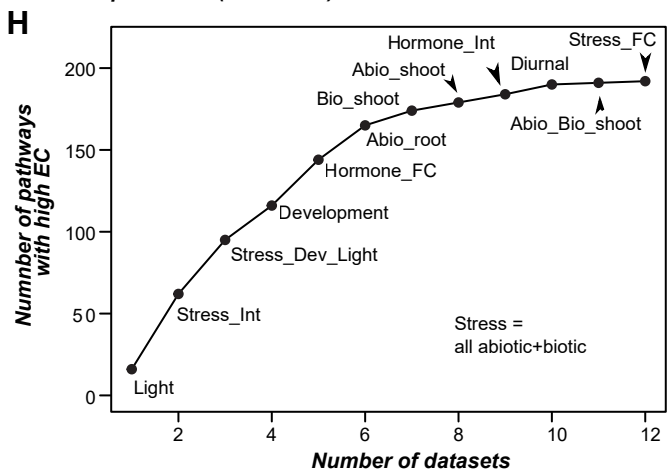

Supplement: S3 Fig — (A) Left panel: Individual and combinations of datasets used to determine pathway EC percentiles. A “+” indicates that the dataset in question was used (either individually or in combination) for the distributions depicted in the Right panel. Right panel: Distribution of randomized pathway EC. Pathway gene membership was randomized 100 times. Median EC per pathway size is shown in this distribution. (B) Relationship between pathway EC percentiles calculated using the combined stress gene expression dataset and those calculated based on one of the individual stress datasets, biotic/shoot. (C) abiotic/root. (D) Relationship between pathway EC percentiles calculated using the light, development and stress combined dataset and those calculated based on individual dataset, development. (E) light. (F) Relationship between pathway EC percentiles calculated using the fold change and absolute intensities for the stress gene expression dataset. (G) Relationship between pathway EC percentiles calculated using fold change and absolute intensities for the hormone gene expression dataset. Dashed line: y = x. Each dot represents a pathway. (H) The change in the number of pathways with high EC (y-axis) with the addition of more expression datasets (x-axis). (PDF) [file pcbi.1005244.s003.pdf]

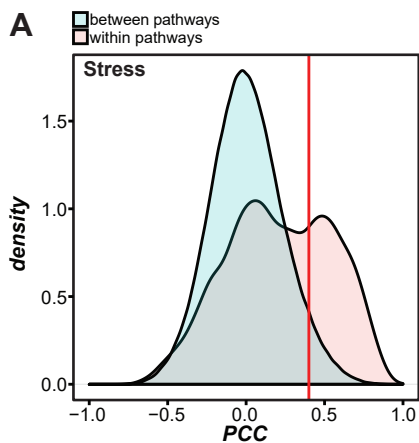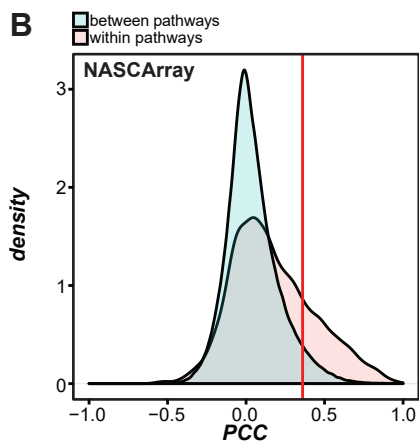

Supplement: S4 Fig — (A) Distributions of PCC values of within pathway gene pairs (light red) and between pathway gene pairs (light blue) using the condition-dependent, stress dataset. Red line: PCC at the 95th percentile of between pathway gene pair PCC distribution. (B) Same as (A) but using the condition-independent, NASCArray dataset. (PDF) [file pcbi.1005244.s004.pdf]

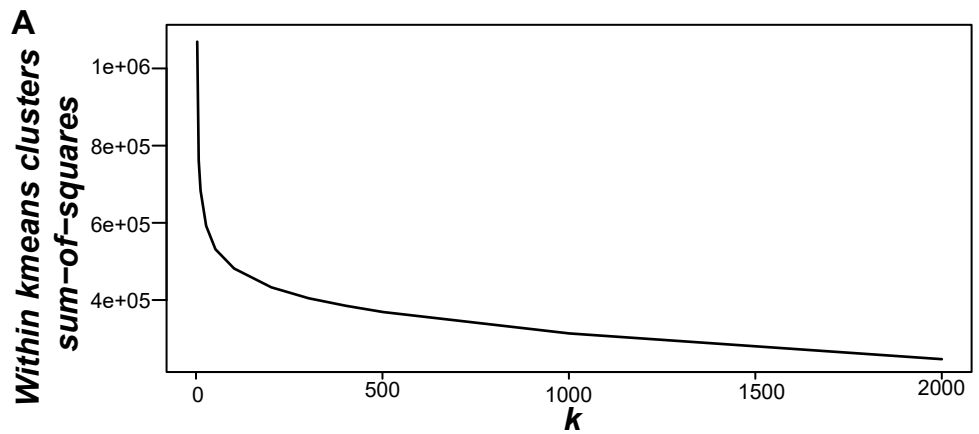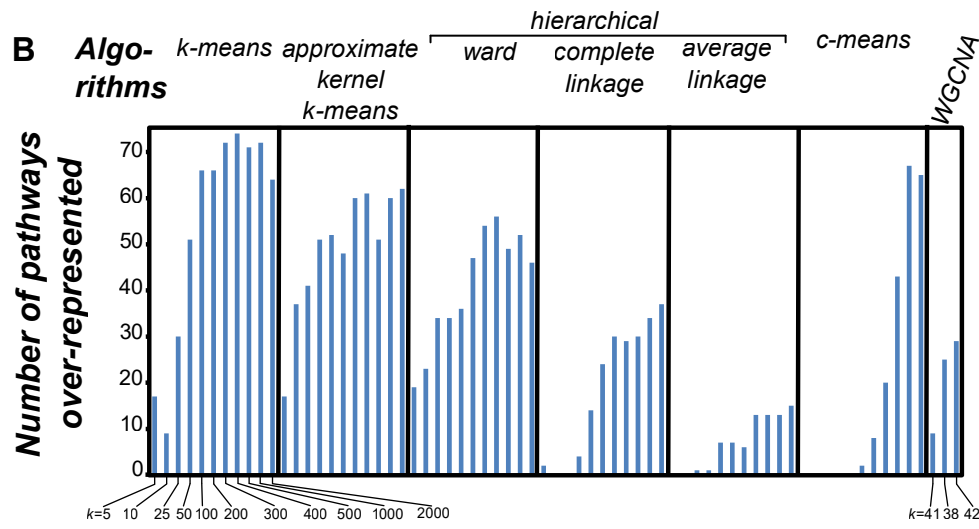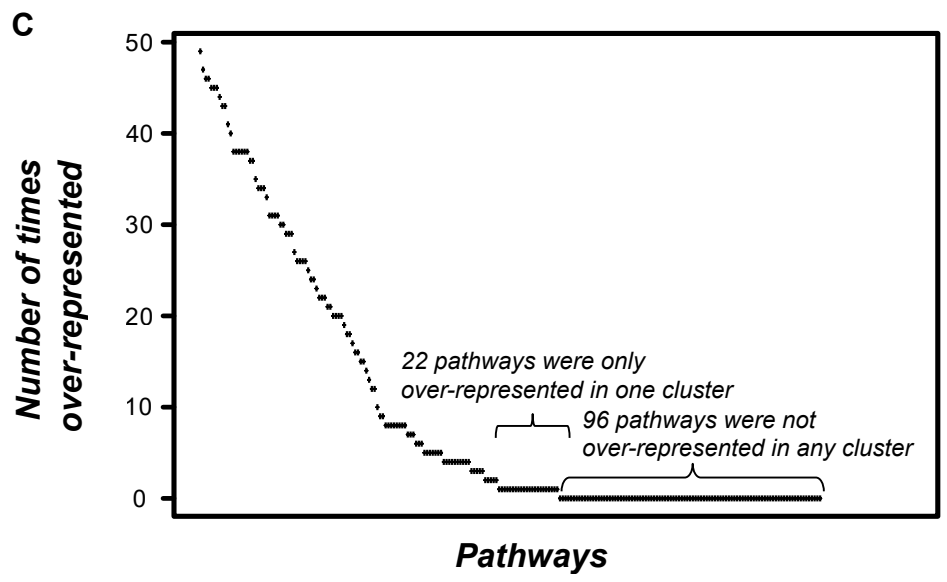

Supplement: S5 Fig — (A) Elbow plot showing within cluster sum of squares for k = 5–2000. (B) The number of pathways over-represented (y-axis) in clusters obtained using different algorithms and cluster numbers (x-axis). (C) Distribution of the number of times that a pathway is over-represented in a cluster (sorted high to low). (PDF) [file pcbi.1005244.s005.pdf]

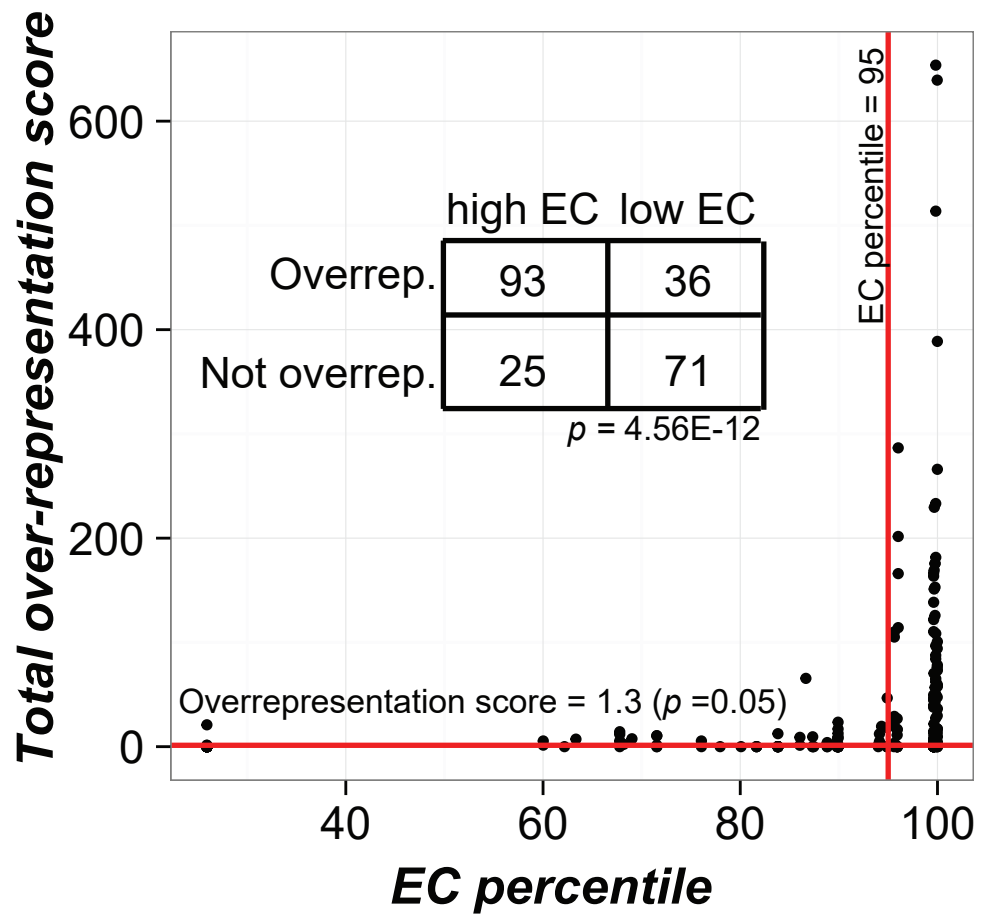

Supplement: S6 Fig — The relationship between the pathway-cluster over-representation score (y-axis) and pathway EC percentile (x-axis). Horizontal red line: over-representation score = 1.3, corresponding to adjusted p-value of 0.05. A pathway is considered over-represented if the over-representation score is >1.3. Vertical red line: EC percentile = 95. High EC pathway has an EC percentile>95. The insert is the contingency table for testing (Fisher’s exact test) whether the number of high EC pathways is higher among over-represented clusters than randomly expected. (PDF) [file pcbi.1005244.s006.pdf]
